# Preconception care by family physicians and general practitioners in Japan

**DOI:** 10.1186/1471-2296-6-31

**Published:** 2005-07-28

**Authors:** Kazuya Kitamura, Michael D Fetters, Nobutaro Ban

**Affiliations:** 1 Department of General Medicine, Nagoya University Hospital 65 Tsurumai-cho, Showa-ku, Nagoya 466-8560 Japan; 2 Department of Family Medicine, University of Michigan Health System 1018 Fuller Street, Ann Arbor, Michigan, USA 48109-0708

## Abstract

**Background:**

Preconception care provided by family physicians/general practitioners (FP/GPs) can provide predictable benefits to mothers and infants. The objective of this study was to elucidate knowledge of, attitudes about, and practices of preconception care by FP/GPs in Japan.

**Methods:**

A survey was distributed to physician members of the Japanese Academy of Family Medicine. The questionnaire addressed experiences of preconception education in medical school and residency, frequency of preconception care in clinical practice, attitudes about providing preconception care, and perceived need for preconception education to medical students and residents.

**Results:**

Two hundred and sixty-eight of 347 (77%) eligible physicians responded. The most common education they reported receiving was about smoking cessation (71%), and the least was about folic acid supplementation (12%). Many participants reported providing smoking cessation in their practice (60%), though only about one third of respondents advise restricting alcohol intake. Few reported advising calcium supplementation (10%) or folic acid supplementation (4%). About 70% reported their willingness to provide preconception care. Almost all participants believe medical students and residents should have education about preconception care.

**Conclusion:**

FP/GPs in Japan report little training in preconception care and few currently provide it. With training, most participants are willing to provide preconception care themselves and think medical students and residents should receive this education.

## Background

Appropriate preconception care provided by family physician/general practitioners (FP/GPs) can provide great benefits to mothers and infants. [1-4] In spite of many potentially helpful interventions prior to conception, there is little literature illustrating the effectiveness of preconception services delivery through primary care settings. Muchowski and Paladine present the evidence of effectiveness for components of preconception care that could be provided in primary care settings.[4] Korenbrot and colleagues conducted a systematic review and found no RCTs of prepregnancy interventions, though one RCT was conducted among women who had a negative pregnancy test.[5] Despite recent research demonstrating the importance of assessing primary care workers views on preconception care,[6] we found no published research on Japanese FP/GPs' approaches to the provision of preconception care to women of reproductive age.

Such research is needed because obstetricians in Japan usually cannot provide preconception counseling. Women infrequently present to OB/GYN physicians prior to conception unless they have gynecological problems. Many of our primary care colleagues consider women's health issues to be outside the realm of their practice, and they lack systematic education in preventive care during family/general medicine training. Despite a longstanding call for family physicians to provide preventive care in Japan,[7] we hypothesized that few FP/GPs are providing preconception counseling.

The best proven preconception intervention, taking folic acid supplements one to two months prior to conception, has been shown to prevent neural tube defects.[8] Unfortunately, many pregnant women do not have their first visit for prenatal care until eight weeks of pregnancy or later, even though fetal development is most vulnerable to development of neural tube defects during this time.[1] Epidemiological studies published over the last fifteen years document that prenatal supplementation with folic acid reduces the risk of neural tube defects, such as spina bifida and anencephaly. [9-11] In many countries, daily consumption of 0.4 mg of folic acid is recommended for reproductive-aged women, and 4 mg is recommended for those who previously had an affected fetus/infant.[12,13] The World Health Organization (WHO) recommends preconception care, including folic acid supplementation, for primary prevention of birth defects in developing and developed countries alike.[14]

Until recently, there has been no recommendation for Japanese women to take a folic acid supplement prior to conception. In December 2000, the Ministry of Health, Labour and Welfare (MHLW) formally recognized the importance of reproductive-aged women taking folic acid supplementation for the prevention of neural tube defects.[15] This recommendation was based on research showing that the rate of neural tube defects is reduced by about 72% when women take folic acid supplementation one to three months prior to conception. The MHLW recommended to the Japan Medical Association, Japan Society of Obstetrics and Gynecology, and Japan Pediatric Society that these organizations should provide their membership with adequate information about the value of taking folic acid to reproductive-aged women.[15]

In addition, there are other compelling topics to cover during preconception counseling based on theoretical considerations and indirect evidence. A particularly important topic in Japan is screening for immunity to rubella. The prevalence of rubella vaccination in Japan is only about 70%.[16] Rubella vaccination is not mandatory due in part to serious side effects that resulted from an MMR vaccination manufactured in Japan several years ago.[16] Antibody negative women can be safely vaccinated prior to pregnancy during preconception care.[17]

Excessive alcohol intake during pregnancy causes fetal alcohol syndrome.[18] Smoking during pregnancy is associated with low birth rate infants.[19] As post-partum hemorrhage is the most important cause of preventable maternal mortality in Japan,[20] prevention of anemia through early detection and treatment with iron supplementation merits consideration. As the average intake of calcium, especially in reproductive women in Japan, is lower than recommended in general, calcium intake is a particularly salient issue for Japanese women of childbearing age. Though controversial, exercise, and mechanisms to optimize pregnancy are among other potentially beneficial effects of preconception counseling. [1-4] Based on clinical experience with a large population of Japanese couples desiring pregnancy, but having difficulty conceiving and not willing to see an infertility specialist, we consider brief counseling on basal body temperature monitoring and timing of intercourse as topics relevant in Japan.

Given the importance of these issues in maternal child health, the purpose of this research was to elucidate the knowledge of, attitudes about, and practices of preconception care for reproductive-aged women by FP/GPs in Japan.

## Methods

In this survey research, we distributed a structured questionnaire to physicians who were registered members of the Japanese Academy of Family Medicine (JAFM). The JAFM has membership based on interest, not criteria such as board certification or completion of family medicine residency training. As family medicine is still a young discipline in Japan, most members have trained in non-family medicine programs. The physician members include a diverse group: those trained in a family medicine training program in the US or Japan, those trained in another specialty or multiple specialties and became a general practitioner after entering practice, and those who trained in a general internal medicine program in Japan or the US.

**Figure 1:**
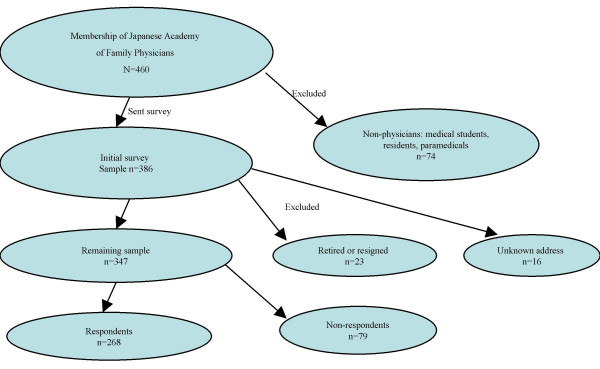
Selection of survey participants.

The JAFM was established in 1986 and has taken a leadership role in establishing family medicine in Japan.[21] The academy had a membership of 460 during the research period. Most members of the JAFM are practicing physicians and/or teachers of family medicine. The membership also includes medical students, residents and paramedical staff who are interested in family medicine. We excluded from the analysis non-physician members, medical students, physicians not in active practice, and those who had resigned from the JAFM (Figure 1).

All participants were asked to complete a questionnaire with items addressing: 1) experiences in preconception education during medical school and residency training; 2) the frequency of providing preconception care in their practice; 3) their attitudes about providing preconception care; and 4) their perceptions of the need for preconception education for medical students and residents. The instrument was distributed with a cover letter requesting their participation. A reminder was sent to non-respondents twice at two-week intervals. A fourth mailing included the final request and a second copy of the instrument.

The components of preconception care we investigated are depicted in Table 1. Some are compelling based on best evidence (such as folic acid supplementation, smoking cessation),[5] while others were chosen based on circumstances particular to Japan as presented above.

**Table 1:** Preconception Care Interventions

Services	Intervention
Folic acid supplementation	Advise 0.4 mg of folic acid daily (4 mg if previous pregnancy with neural tube defect) three months prior to conception.
Smoking cessation	Educate about the risks of smoking during pregnancy and counsel about smoking cessation.
Anemia screen	Check hematocrit/CBC and recommend iron supplement if anemia is detected.
Testing for rubella antibody	Check IgG rubella antibody before conception. If the test is negative, vaccinate and avoid conception for three months.
Alcohol restriction	Screen for alcoholism by using a validated questionnaire, and counsel or refer if positive screen.
Restricting caffeine	Restrict caffeine intake to less than 250 mg a day.
Exercise	Advise regular to moderate exercise before and during pregnancy.
Calcium supplementation	Assess calcium intake and as needed supplement for a target of 1200 mg daily.
Use of basal body temperature*	Conduct basal body temperature every morning to identify the day of ovulation.
Timing of intercourse*	Advise intercourse every other day around time of ovulation to maximize chances of conception.

*For couples having difficulty conceiving

We analyzed the data using SPSS (Statistical Package for Social Sciences). Simple statistics were calculated on the demographics data. Statistical significance of continuous measures was tested by two-tailed student t-test, and categorical data were tested by the chi-square test where appropriate. Physicians who reported their specialty as family medicine were compared for differences with physicians who reported their specialty as general internal medicine.

## Results

Of the 460 members of the JAFM during the study period, we excluded the 74 non-physician and medical student members from the survey. Of the 386 physicians to whom we distributed instruments, 291 physician members responded. Sixteen letters were returned because of unknown addresses. We dropped 23 physicians from the analysis who reported that they were retired or had resigned from the academy. Thus, 268 of 347 eligible physicians (response rate 77.2%) were included in the analysis (Figure 1). The demographics of the participants are listed in Table 2. Most were men (86%) and the mean age was almost 40 years. Most physicians reported seeing reproductive-aged women in their offices.

**Table 2:** Participant Demographics (n = 268)

	n	(%)
Gender		
Male	230	(85.8)
Age		
Mean (range)	40.1 (25–73)	
Period after graduating from med school (yrs)		
Mean (range)	14.4 (1–26)	
Specialty (total response = 308)*		
General Internal Medicine	140	(45.5)
Family Medicine	102	(33.1)
Specialty in Internal Medicine	19	(6.2)
General Medicine	10	(3.2)
Surgery	8	(2.6)
Pediatrics	8	(2.6)
Psychiatry	6	(1.9)
Orthopedics	5	(1.6)
Others	10	(3.2)
Experiences of out-patient training in		
Pediatrics	160	(59.7)
OB/GYN	99	(36.9)
Average number of patients per week		
All	142 /wk	
Reproductive women	11 /wk	
Average number of patients by age		
Child (0–15)	15.3	(8.9)
Adolescence (16–19)	9.5	(7.0)
Adult (20–64)	43.9	(35.0)
Elderly (65 and more)	69.5	(49.4)

*multiple responses were possible

With regard to educational experiences in preconception counseling during medical school and residency training (Table 3), most participants reported they had received little. The most common relevant training they reported receiving included: smoking cessation (71%), screening for anemia (64%), and blood testing for rubella antibody (58%). Few reported training experiences in providing folic acid supplementation (12%), timing of intercourse to maximize chances of conception (14%), or exercise during pregnancy (18%).

**Table 3:** Japanese family physicians' experiences in preconception education during medical school and residency training (n = 268)

	n	(%)
Smoking cessation	191	(71.3)
Testing for anemia	172	(64.2)
Blood testing for rubella antibody	156	(58.2)
Use of basal body temperature monitoring	125	(46.6)
Restricting alcohol intake	120	(44.8)
Increasing calcium intake	86	(32.1)
Restricting caffeine intake	52	(19.4)
Exercise during pregnancy	48	(17.9)
Timing of intercourse to maximize chances of conception	38	(14.2)
Folic acid supplementation	31	(11.6)

Preconception care practices of the respondents are depicted in Table 4. Many participants reported often or always addressing smoking cessation with reproductive-aged women (60%). Some reported they provide screening for anemia (35%) and counseling about restricting alcohol intake (27%). Few reported they addressed either calcium intake through foods/supplements (10%), or folic acid supplementation (4%).

**Table 4:** Japanese family physicians' self-reports of preconception care in their clinical practice (n = 268)

	Never/Almost Never		Sometimes		Often/Always		No Response	
	
	n	(%)	n	(%)	n	(%)	n	(%)
Timing of intercourse to maximize chances of conception	222	(82.8)	20	(7.5)	9	(3.6)	17	(6.3)
Folic acid supplementation	217	(81.0)	24	(9.0)	11	(4.1)	16	(6.0)
Exercise during pregnancy	198	(73.9)	32	(11.9)	20	(7.5)	18	(6.7)
Testing for rubella antibody	180	(67.2)	55	(20.5)	21	(7.8)	12	(4.5)
Increasing calcium intake	168	(62.7)	61	(22.7)	27	(10.1)	12	(4.5)
Restricting caffeine intake	166	(61.9)	55	(20.5)	31	(11.6)	16	(6.0)
Use of basal body temperature monitoring	157	(58.6)	60	(22.4)	38	(14.2)	13	(4.9)
Restricting alcohol intake	107	(39.9)	74	(27.6)	72	(26.9)	15	(5.6)
Testing for anemia	56	(20.9)	112	(41.8)	94	(35.1)	6	(2.2)
Smoking cessation	37	(13.8)	63	(23.5)	162	(60.4)	6	(2.2)

Their attitudes about providing preconception care are depicted in Table 5. About two thirds of participants reported their willingness to provide preconception care about such topics as calcium intake (70%), blood testing for rubella antibody (69%), and restricting caffeine intake (64%). On the other hand, some expressed dissatisfaction with counseling about timing of intercourse to maximize chances of conception (46%), and use of basal body temperature monitoring (22%). More than 60% reported their willingness to provide folic acid supplementation, though one in four stated they would not provide it. Almost all participants think medical students (95%), and residents (91%), should have education in preconception care.

**Table 5:** Japanese family physicians' willingness to provide preconception care in their practice (n = 268)

	Currently Provide		Willing to Provide		Would Not Provide		No Response	
	
	n	(%)	n	(%)	n	(%)	n	(%)
Smoking cessation	154	(57.5)	103	(38.4)	5	(1.9)	6	(2.2)
Screening for anemia	103	(38.4)	145	(54.1)	11	(4.1)	9	(3.4)
Restricting alcohol intake	90	(33.6)	143	(53.4)	26	(9.7)	9	(3.4)
Use of basal body temperature monitoring	61	(22.8)	137	(51.1)	59	(22.0)	9	(4.1)
Restricting caffeine intake	42	(15.7)	171	(63.8)	42	(15.7)	13	(3.8)
Blood testing for rubella antibody	41	(15.3)	186	(69.4)	30	(11.2)	11	(4.1)
Increasing calcium intake	37	(13.8)	18	(70.1)	34	(12.7)	9	(3.4)
Exercise during pregnancy	30	(11.2)	165	(61.6)	57	(21.3)	16	(6.0)
Folic acid supplementation	14	(5.2)	170	(54.1)	68	(25.4)	16	(6.0)
Timing of intercourse to maximize chances of conception	14	(5.2)	114	(42.5)	122	(45.5)	18	(6.7)

Though the instrument did not have open-ended questions, a number of respondents (n = 69, 26%) provided comments indicating they had been unaware of the recommendation for women to take folic acid supplementation and they were pleased to learn from the survey about this important issue. In contrast, a few (n = 11, 4%) stated they did not understand why FP/GPs should provide preconception care.

There were no statistical or clinically meaningful differences between the reports of physicians who reported their specialty as family medicine and physicians who reported their specialty as general internal medicine.

## Discussion

The movement to establish family medicine in Japan started at least 20 years ago.[7] There has been much debate about whether Japanese family physicians should provide obstetric and other women's health care. There are few FP/GPs who provide OB care in Japan.[22] Our study reveals that few FP/GPs have educational experiences in the provision of preconception care, and few actually provide this care in their practices. However, it also reveals their willingness to provide preconception care in the future after appropriate educational experiences.

Japanese FP/GPs seem reluctant to inquire about human sexuality issues.[23] In the current study, some reported they could not ask patients about future pregnancy plans during a routine acute visit. Our results show FP/GPs in Japan are not accustomed to addressing preconception-related topics (timing of intercourse, folic acid supplementation, exercise during pregnancy), while they are familiar with more general topics (smoking cessation, screening for anemia, calcium intake). FP/GPs are in the unique position to provide health care services to male and female patients of all ages,[24,25] and they have many opportunities to discuss patients' concerns. For example, young parents do not often visit family physicians for their own health problems, but do come for their children. At these visits, family physicians can also discuss family planning. For this reason, FP/GPs in Japan need training in women's health care, even if they will not provide deliveries in the future. We hope these data will provide a catalyst for dialogue among Japan's FP/GPs about regular provision of preconception counseling.

Remarkably, only 10 % of participants reported knowledge of folic acid supplementation and few reported providing this care. Yamanaka surveyed pregnant women about the importance of folic acid.[26] This research revealed that only 8% reported they knew well about its importance, and 46% stated they did not know at all. Furthermore, the small percentage of participants who knew well about the importance of folic acid reported that they learned it from a newspaper, TV, or magazine, while only 16% learned it from medical professionals. Based on these data, Yamanaka emphasized that medical professionals should provide correct and concise information about folic acid.

As the MHLW only recently recommended folic acid supplementation to reproductive-aged women,[15] it is not too surprising that many FP/GPs do not know the importance of folic acid and few provide this care. The JAFM was not included in the list of organizations notified of the MHLW policy change. Based on these data it is clear that its membership is interested in women's health issues and should be included in notifications about women's health policy changes formulated at the government level.

About 70% of participants reported they are willing to screen women for rubella antibody, though almost 70% reported not providing this care in their practice. Given the historic mistrust of the rubella vaccine and the low rate of vaccination in Japan,[16] Japanese family doctors need to proactively address the topic. The lack of mandatory vaccination is a loophole in public health policy[16] and highlights why screening women of childbearing potential for rubella antibody is especially important. Delay in testing, and hence immunization, leads to an increased risk for congenital rubella, a highly serious disease. Non-immune pregnant women should post-pone rubella vaccination until after delivery.

Japan's FP/GPs must learn to provide preconception counseling in order to close this important gap in women's health. Fortunately, many of these respondents are willing to provide some level of preconception care even though they currently are not – presumably due in part to a lack of educational experience. Japan's FP/GPs need educational materials and clinical tools to encourage women to make an office visit and receive preconception care. Family medicine training in Japan heavily emphasizes adult and geriatric medicine with little emphasis on prenatal, newborn, children, adolescent, or women's health.[7,21,27] Initial efforts to disseminate information about preconception care are underway through the medical literature,[25] though other educational efforts will no doubt be needed. Data from Japanese women from Japan who are on temporary assignment in the United States, illustrate low levels of knowledge about prenatal folic acid supplementation and resistance to take supplements.[28]

A potential limitation of this study is selection bias. The participants are limited to physician members of the JAFM and the data might not reflect the current situation of all FP/GPs in Japan. This bias would likely favor the most motivated physicians and the estimates herein probably represent the upper limits of willingness to provide preconception care by the population of primary care providers in Japan. If this interpretation is correct, the need for public campaigns about the importance of preconception care and training of Japan's family physicians are even more imperative.

Despite compelling evidence of the effectiveness of folic acid supplementation and other preconception care for the reduction of serious birth defects at a very low cost, these data provide evidence that the WHO's message about the importance has not filtered down to the clinical level even in a developed country like Japan. Continued efforts to spread and diffuse the WHO's message are desperately needed for the advancement of maternal-child health.

## Conclusion

Our study reveals that many Japan's FP/GPs have limited training in preconception care and few currently provide it. Most participants report their willingness to provide preconception care themselves and educational campaigns are needed to enhance preventive care provided by FP/GPs in Japan.

## List of abbreviations used

CBC – complete blood count

FP/GPs – family physicians and general practitioners

IgG – immunoglobulin G

JAFM – Japanese Academy of Family Medicine

mg – milligrams

MHLW – Ministry of Health, Labour and Welfare

OB/GYN – obstetrics and gynecology

RCT – randomized controlled trial

SPSS – Statistical Package for the Social Sciences

WHO – World Health Organization

wk – week

## Competing interests

The author(s) declare that they have no competing interests.

## Authors' contributions

KK contributed to the conception and study design, performed data analysis, interpretation, and draft the manuscript. MDF contributed to the conception and study design and critical revision of the manuscript. NB participated in the study design and critical revision of the manuscript. All authors read and approved the final manuscript.

## Pre-publication history

The pre-publication history for this paper can be accessed here:


